# UHPLC-MS/MS Analysis of Antibiotics Transfer and Concentrations in Porcine Oral Fluid after Intramuscular Application

**DOI:** 10.3390/ph15020225

**Published:** 2022-02-14

**Authors:** Anna Gajda, Ewelina Nowacka-Kozak, Małgorzata Gbylik-Sikorska, Piotr Cybulski

**Affiliations:** 1 Department of Pharmacology and Toxicology, National Veterinary Research Institute, Partyzantow 57, 24-100 Pulawy, Poland; ewelina.nowacka@piwet.pulawy.pl (E.N.-K.); malgorzata.gbylik@piwet.pulawy.pl (M.G.-S.); 2 Goodvalley Agro S.A., Dworcowa 25, 77-320 Przechlewo, Poland; piotr.cybulski.dvm@gmail.com

**Keywords:** antibiotics, food safety, oral fluid, pigs, UHPLC-MS/MS

## Abstract

The monitoring of antibiotic use in animals is a crucial element to ensure food safety. The main goal of this study was to analyse the distribution of selected antibiotics to porcine oral fluid, as well as to demonstrate that an oral fluid is an alternative to other biological matrices used in the control of antibacterials. Therefore, an animal study with pigs treated using seven different antibiotics was performed. Sulfadoxine (SDX) with trimethoprim (TRMP), lincomycin (LIN), tiamulin (TIAM), tylosin (TYL), amoxicillin (AMX) and penicillin G (PEN G) were injected intramuscularly to pigs, and concentrations of these analytes in the oral fluid were assessed. Ultra-high-performance liquid chromatography coupled with mass spectrometry (UHPLC-MS/MS) was used to quantify the analytes. On the first day of medication, the highest concentrations for SDX and TRMP at the level of 22,300 µg/kg and 14,100 µg/kg were found, respectively. The concentrations of LIN (10,500 µg/kg) and TIAM (7600 µg/kg) were also relatively high. The peak of TYL was recorded on the second day of drug administration. Most of the analytes were present in oral fluid for 30 days, apart from TYL, which was detected for 27 days. It was found that AMX and PEN G were quantified only for 5 and 8 days, respectively, at very low concentrations. It was found that oral fluid can be used for the verification of antibiotics on pig farms.

## 1. Introduction

Antimicrobial residues are of food safety concern. The residues of drugs may result in many biological adverse effects and allergic reactions in consumers, as well as the spread of drug-resistant bacteria and bacterial resistance acquisition [1]. Consolidation of pig production requires ensuring adequate conditions for the maintenance of animals with high health status, consistent with the guidelines for animal welfare [2,3]. At the same time, the control of antibiotics in animals is an important element providing the high quality of pig farming, as well as the protection of consumers. In the official residues monitoring programs implemented in the Member States of the European Union, antibiotics are mainly inspected in tissues, collected from animals at the slaughterhouses. However, pigs should be tested for potential antimicrobial residues on a farm because postmortem analysis does not enable the monitoring of antibiotics administration during rearing.

It is possible to determine drugs level in blood plasma; however collection of this type of material is associated with exposure of animals to stress, as well as considerable inconvenience for the person taking the samples. The pig’s safety, stress reduction, time-saving, and material collection convenience, determine different sampling techniques. Even though urine and milk samples can be successfully utilised as matrices for the detection of drugs in animals, the first material in question is used solely for the analysis of banned compounds, whereas usage of the latter is limited to cows only [4]. In the antemortem drugs analysis, drinking water and feed are generally used, but it gives information about the source of contamination, not about the presence of residues in animals.

There are many benefits of using oral fluid in the control of antimicrobial residues compared to matrices used so far. Collecting oral fluid is animal-friendly and noninvasive, devoid of any needles or snout loop [5]. The technique of sampling requires the use of cotton rope, which is bitten and chewed to reduce the stress of animals. Oral fluid can be collected from a single animal, but it is usually collected from a group of animals, which offers the time and cost-effective approach [6]. A large population of pigs can be checked on the presence of antibacterials by taking one bulk sample, as one rope should be sufficient for even 40–50 pigs. The cost of analysing one bulk sample is significantly reduced compared to testing hundreds of individual samples, as in plasma analysis.

Oral fluid was demonstrated as a matrix in the evaluation of some metabolic diseases in humans by detecting a variety of analytes in the olden days [7]. After these early reports, developments in oral fluid diagnostics were generally obfuscated by improvements in the detection of some compounds in blood or serum [8]. Nevertheless, in the 1990s, intensive research on the oral fluid-based assays as a medium for infectious and non-infectious diseases, drugs, hormones and disease marker detection in humans were started and implemented again [9,10,11]. In veterinary medicine, significant research was carried out for the detection of many viral infections [12,13]. Literature data demonstrate a high probability of drug transmission after their administration [14]. However, very limited information about the detection of antibiotics or other compounds in oral fluid is presented. Recently, there were a few reports presenting the determination of some antibacterials such as ceftiofur and oxytetracycline in swine oral fluid [15]. In that study, both compounds were only qualitatively determined as positive or negative, using an ELISA test. In another work, described by Oruc et al., 2013, the application of a biochip array-based immunoassay in the detection of some antimicrobials in oral fluids was demonstrated, but this technique works only for clean samples collected under controlled research [16]. A suitable analytical method is an essential analytical tool in the determination of drugs in the matrix of interest. Therefore, in the paper of Gajda et al., 2017, a liquid chromatography–tandem mass spectrometry (LC-MS/MS) method for the determination of oxytetracycline and 4-epi oxytetracycline in swine oral fluid after intramuscular injection was reported [17]. The results of this study demonstrate that the tested material from pigs seems to be an effective tool for the control of proper medical treatment and residues prevention. However, to prove that oral fluid collected from medicated pigs is an appropriate matrix for the non-invasive detection of antimicrobials, more analytes should be tested by an accurate and sensitive method.

In this paper, the transfer and concentrations of seven different antibiotics in swine oral fluid, commonly used in pigs, were studied for the first time. In six separated experimental groups, animals were therapeutically treated with a veterinary product containing sulfadoxine (SDX) with trimethoprim (TRMP), lincomycin (LIN), tiamulin (TIAM), tylosin (TYL), amoxicillin (AMX), and penicillin G (PEN G), one drug per group. The aim of this study was to develop and validate a multiresidue method for the simultaneous determination of seven antibiotics as well as demonstrate an oral fluid as an alternative to other biological matrices used so far in the control of antibacterials.

## 2. Results

### 2.1. Validation of an Analytical Method

**Figure 1:**
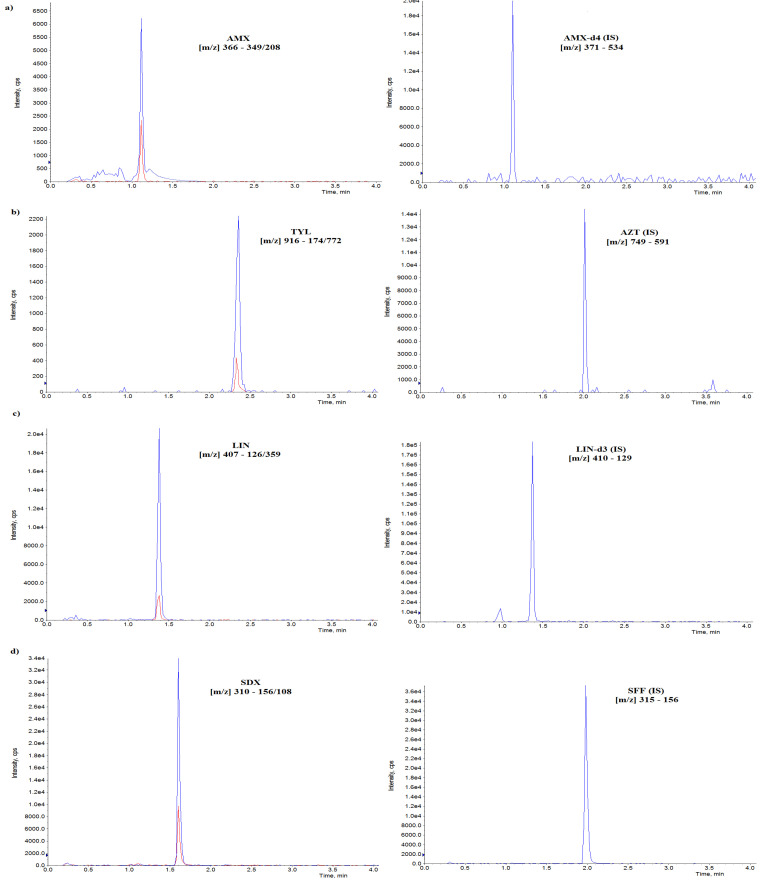

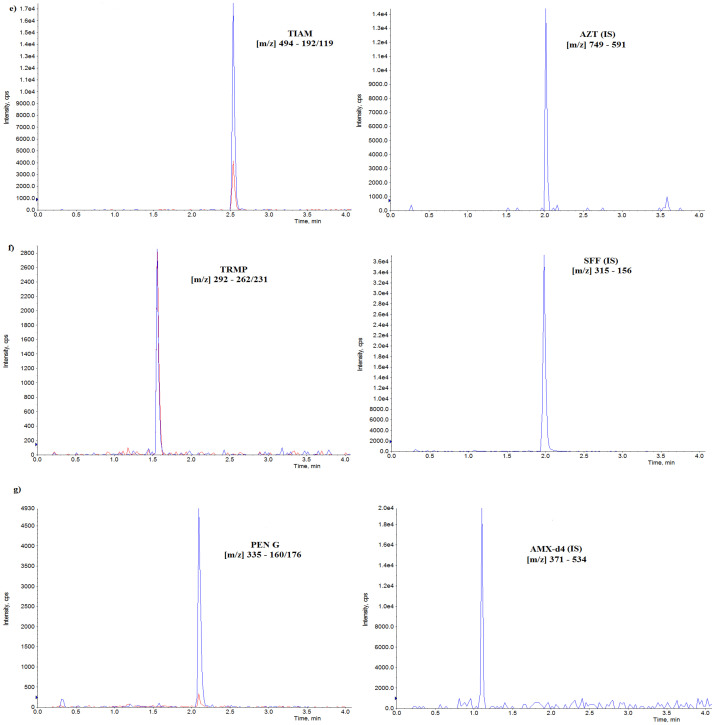
Chromatograms of oral fluid samples spiked at validation level (VL) with analysed compounds and corresponding internal standards (IS), (**a**) AMX—amoxicillin, (**b**) TYL—tylosin, AZT—azithromycin, (**c**) LIN—lincomycin, (**d**) SDX—sulfadoxine, SFF—sulfaphenazole, (**e**) TIAM—tiamulin, (**f**) TRMP—trimethoprim, (**g**) PEN G—penicillin G.

The UHPLC-MS/MS method used in this research for the determination of seven different compounds in oral fluid was fully validated. All matrix-matched calibration curves showed good linearity (r^2^ > 0.997) for all analytes. The coefficients of variation (CVs, %) for repeatability, were lower than 10% and below 15% for within-laboratory reproducibility. The selectivity testing allowed us to verify that no peaks from endogenous compounds were detected in the retention time corresponding to each analyte or internal standard. The average recoveries were in the range of 87.6–107%, depending on the analyte. The method was satisfactorily sensitive with limits of quantification (LOQs) established in the range of 2–10 µg/L, whereas limits of detection (LODs) were in the range of 1–5 µg/L. The results are shown in Table 1. The chromatograms of oral fluid samples spiked at validation level (VL) with all analysed compounds are presented in Figure 1.

### 2.2. Detection and Quantification of SDX, TRMP, TYL, TIAM, LIN, AMX and PEN G in Oral Fluid Samples

For the quantitative analysis of SDX, TRMP, TYL, TIAM, LIN, AMX and PEN G, an equation from the regression analysis of the matrix-matched calibration curve was used, and the corresponding internal standards were used: SFF for SDX and TRMP, AZT for TYL and TIAM, LIN-d3 for LIN, AMX-d4 for AMX and PEN G. Before the start of the experiment, all pigs were checked to be negative for all tested substances. After intramuscular injection of medicines according to therapeutic indications: two doses with 48h interval for SDX + TRMP and AMX, and three doses with 24 h interval for TYL, TIAM, LIN, PEN G, all compounds were detectable in oral fluid. However, significant differences in concentration on the first day were observed. The highest concentrations for SDX and TRMP at the level of 22,300 µg/L and 14,100 µg/L was found, respectively. The concentrations of LIN (10,500 µg/L) and TIAM (7600 µg/L) were also relatively high on the first day of medication. The peak of TYL was recorded on the second day of drug administration, but concentrations in the oral fluid were much lower from the abovelisted compounds. It was found that AMX and PEN G were poorly passed to oral fluid, and the level of these analytes was determined as 11.3 µg/L for AMX and 93 µg/L for PEN G. The concentrations of antimicrobials at each time point are listed in Table 2. The content of analytes decreased gradually over time. After three days of treatment, the highest concentration of 4010 µg/L for SDX was recorded. The levels of LIN and TIAM after the finishing of drugs administration were similar, with concentrations of 1550 µg/L for LIN and 1560 µg/L for TIAM. The sampling took 30 days, and after this time, SDX + TRMP were detected at the level of 23.0 µg/L and 22.4 µg/L, respectively. Slightly higher concentrations were recorded for LIN and TIAM (58.1 µg/L and 46.9 µg/L, respectively) on the 30th day. TYL persisted a little less and the 27th day was the last day with a quantified concentration. In contrast, PEN G was present in oral fluid for 8 days, while AMX persisted for only 5 days. Different withdrawal periods for each antimicrobial were established. The length of withdrawal periods for each drug used in the experiment are shown in Table 3, while the concentrations found at this time are presented in Table 2. At a withdrawal period of 8 days for SDX + TRMP, the concentrations were determined as 247 µg/L and 67.1 µg/L, respectively. The highest level at withdrawal time for LIN—437 µg/L was recorded. Low concentration for TYL at this time point was found (6.6 µg/L), while AMX and PEN G were below the limit of quantification of the applied analytical method.

## 3. Discussion

The antimicrobials overused in domestic animals can lead to residues in tissues and other animal products intended for human consumption. In some cases, the lack or inadequate cleaning of water supply systems or feed dispensers after previous treatment can also give positive results. Apart from the risk to public health, the presence of antibiotics in tissues often leads to the removal and destruction of a significant quantity of meat, which contributes to a serious economic consequence. Therefore, a new method for the control of antibacterials during rearing is strongly needed. The fast and simple sampling of oral fluid with the cotton rope is an effective tool for the monitoring of reasonable treatment. In case of positive oral fluid analysis results, appropriate measures can be taken to prevent the slaughtering of positive animals intended for sale in order to avoid huge costs connected with the disposal and utilization of contaminated meat.

For the analysis of antibacterials in oral fluid, a UHPLC-MS/MS analytical method was developed. The extraction used in the presented method allows for simultaneous analyses of seven different compounds from six various chemical groups. Additionally, the simple and fast sample preparation, as well as a cleanup step by filtration, give an opportunity to test a lot of samples in a short time. The paper described a biochip array-based immunoassay method for the detection of six different antimicrobial drugs, tetracyclines were very poorly recovered in dirty samples (5%), and for other drugs (norfloxacin, ceftiofur and florfenicol), recovery values were much higher than 100% [16]. This means that the presented technology with a biochip array-based immunoassay can work only for clean oral fluid samples and is of value if it is being used under typical field conditions. Field oral fluid samples are reflective of the environment in which pigs are housed. Therefore, it was necessary to develop such a method enabling the analysis of all compounds with sufficient sensitivity, accuracy and precision with reliable recovery values. In our previous research, we tested the passage and concentrations of oxytetracycline after i.m. injection [17]. For that purpose, a sensitive LC-MS/MS method was developed with satisfactory validation results. That experiment proved that oxytetracycline is present in oral fluid for a long time after medication. At the withdrawal period (21st day), the concentration was 30.8 µg/kg in the group where all pigs were treated and 11.6 µg/kg in the group where the antibiotic was administered to half of the animals in one pen. However, in the present study, the experiment with seven other compounds was performed for the first time.

In the described work, oral fluid was tested for the detection of some antibacterials for food safety applications. To demonstrate the validity of this matrix, seven drugs from various groups of antibacterials with different chemical and therapeutic properties were administered by intramuscular injection. The compounds tested in this experiment were selected based on a review of the use of antimicrobial drugs used in swine [18]. The route of medicine application is an important element in this research. In pigs treated with medicated feed or water, the presence of drugs can be expected in saliva for most drugs. However, some reports described that β-lactam antibiotics are neutralized by different enzymes present in saliva [19]. Therefore, in our study, AMX and PEN G, which belong to this group of antibacterials, were chosen in the experiment to check the penetration of these substances and analyse the matrix after intramuscular (i.m.) injection. The value of sulphonamides as single antimicrobial agents was greatly diminished both by widespread acquired resistance and by their relatively low potency. However, when combined with diaminopirymidynes such as trimethoprim, their usefulness was enhanced thus SDX with TRMP were applied.

Oral fluid is a mixture of saliva, oral mucosal transudate and gingival crevicular fluid [20]. Antimicrobials can be transported from blood to saliva by simple diffusion and/or active transport [11,21,22,23]. Factors such as lipid solubility, molecular size, degree of ionization of the drug molecule, as well as the effect of salivary pH and the degree of protein binding of the drug, determine the drug availability in oral fluids [24]. Concerning antimicrobials that are weak bases, lipophilic compounds and small molecules diffuse more easily and may reach high concentrations in saliva [24]. SDX and TRMP, which are relatively small molecules (310.33 g/mol and 290.32 g/mol, respectively), reached the highest concentrations in oral fluid at 24 h post-treatment (22,300 µg/L for SDX and 14,100 µg/L for TRMP), while the level of TYL, with molecular weight at 916.1 g/mol, was quantified at 396 µg/L at the same time. The results of our study demonstrate that high lipid-soluble compounds diffuse more easily and reach higher levels in oral fluid. SDX and TRMP are lipophilic compounds, similar to TYL, TIAM and LIN, which are high lipid-soluble substances, and the result of these substances was found greater than for hydrophilic AMX or high water-soluble PEN G.

The pharmacokinetics properties determine, to some extent, the distribution of drugs in many structures of the organism. The results of our research demonstrate the strong relationship between the pharmacokinetics and penetration to oral fluid for most of the analysed substances. Concentrations of most compounds presented in the experiment were high and reached great levels in oral fluid. Most of the analysed antibiotics are basic compounds with relatively high volumes of distribution. LIN is a basic compound with pKa values of about 7.6. It has high lipid solubility and consequently a large apparent volume of distribution. LIN is widely distributed in many fluids and tissues. This antibiotic diffuses across the placenta in many species, and the apparent volume of distribution is >1 L/kg [25]. For TIAM, which is a weak base, pKa 7.6, high levels in the oral fluid were observed, similar to LIN [25]. TYL, as a weak base, pKa 7.1, with a good volume of distribution, reaches high concentrations in many distribution spaces, e.g., in kidneys, lungs, spleen, liver or milk, as well as oral fluid, where good distribution of this macrolide antibiotic was observed [26].

PEN G, for which concentrations in the oral fluid were very low, is an organic acid with pKa 2.7. The group of penicillins have relatively small apparent volumes of distribution (0.2–0.3 L/kg) and short half-lives (0.5–1.2 h) in all species of domestic animals [27]. Acid hydrolysis in the stomach limits the systematic availability of most penicillins from oral preparations. After absorption, they are widely distributed in the extracellular fluid of the body but cross biologic membranes slightly since they are ionized and poorly lipid-soluble [28]. However, for SDX, a weak organic acid with a pKa value of 6.16, the concentrations in the oral fluid were high and lasted for a long time. Most sulfonamides exist predominantly in a non-ionized form in biologic fluids with a pH lower than their pKa [29].

The transport of some drugs to the oral fluid can also be determined by the degree of protein binding. It was observed that compounds with high protein binding show a greater ability for diffusion to saliva. Sulfonamides and diaminopyrimidines, which are generally highly bound to proteins in serum (60–90%), as well as lincosamides, in which serum protein binding is 72%, reached high concentrations in oral fluid [25,29]. Substances with a low binding degree to plasma proteins, i.e., TYL < 50% and AMX ≈ 25%, penetrate to oral fluid in lesser extent [28,30].

## 4. Materials and Methods

### 4.1. Animal Experiment and Sample Collection

All animals were born in the sow farm (8000 DanBred sows) located in Northern Poland, after crossing ♀(♂Landrace × ♀Yorskshire) × ♂Duroc. Piglets were weaned after 4 weeks of lactation with an average weight of 6 kg and then moved to the weaner stable (23,000 weaners). After the next 7 weeks, animals were transported to the 9000-places fattening farm with an average body weight of 28 kg, where the trial was performed. All farms in the aforementioned three-phase model used weekly batches and an all-in all-out system. Specialist veterinary and production management care were provided in each location.

For the purpose of the study, 240 immunologically castrated boars were divided into 8 groups, 30 animals in each pen. For the control group, 30 pigs were used in separate pens. Their initial body weight was between 40 and 60 kg. Pigs were housed at 20–25 °C. Fatteners were fed ad libitum with antimicrobial-free water and feed provided by feeders throughout the study. Before the experiment, water and feed were checked for antimicrobials, and no antimicrobial treatments were administered to these pigs before. All animals in one pen were treated due to the same health issue using disposable needles and the same drug and dosage. Pens were separated from each other with concrete walls. The drugs and dosage administered to each experimental group with an indication of withdrawal period for tissues are listed in Table 3. The oral fluid samples were collected using three cotton ropes (70 cm length with 1 cm diameter) in each pen suspended separately for 25 min on the front wall of the pen with bottoms at the pigs’ shoulder height, away from water and feed.

The research material was one pooled sample of oral fluid from 10 pigs, in triplicate, per pen. Ropes were removed immediately after collection. The first oral fluid sample was collected 24 h after the first injection. The collection of each subsequent sample took place at the same time (11:30 A.M.) for 35 consecutive days. Additionally, oral fluid samples were collected from pigs not treated with any antibiotics. Oral fluid from each pen was pooled into one 50-mL Falcon plastic tube and centrifuged at 9447× rcf for 15 min. Centrifuged material was transferred to a 15 mL plastic tube and frozen at −20 °C until analysis.

### 4.2. Quantitative Analysis by UHPLC-MS/MS

#### 4.2.1. Reagents and Chemicals

All reagents used were of analytical grade. Reference standards of SDX, TRMP, LIN, TIAM, TYL, AMX and PEN G were used for the analysis and quantification of the analytes. Lincomycin-d_3_ (LIN-d_3_), amoxicillin-d_4_ (AMX-d_4_), sulfaphenazole (SFF) and azithromycin (AZT) were used as internal standards. All standards were manufactured and obtained from LGC Standards (Teddington, Middlesex, UK). Formic acid was from Sigma Aldrich (St. Louis, MO, USA). Acetonitrile was obtained from J.T. Baker (Deventer, The Netherlands). Heptafluorobutyric acid was from Fluka (St. Louis, MO, USA). Syringe 0.22 μm Hydrophilic Polyvinylidene Fluoride (PVDF) Membrane Filters were from Restek (College, PA, USA).

#### 4.2.2. Preparation of the Standard Stock Solutions and Working Solutions

Individual stock standard solutions (1000 µg/mL) for AMX, AMX-d4, and PEN G were prepared in ultrapure water and stored in polypropylene vessels. LIN, LIN-d3, TYL, TIAM, AZT, SDX, TRMP and SFF stock standard solutions (1000 µg/mL) were dissolved in methanol. All individual stock standard solutions were stable for at least 6 months when retained in a dark place at −18 °C. A working solution for each analyte was prepared from stock solutions by dilution in ultrapure water and stored at 4–8 °C for 1 month.

#### 4.2.3. Extraction and Cleanup

Before the analysis, the oral fluid samples were centrifuged at 9447× rcf. Next, an aliquot (1 mL) of oral fluid was placed into a 2 mL polypropylene centrifuge tube, and the 30 µL of the internal standard at the concentration of 2 µg/mL was added. For the extraction of analysed antibiotics, 600 µL of 0.5% formic acid was added, vortexed for 30 s and centrifuged for 10 min at 9447× rcf. Then, the supernatant was filtered through a 0.22 mm PVDF filter into an amber glass vial before chromatographic analysis.

#### 4.2.4. LC-MS/MS Analysis

The analysis of SDX, TRMP, LIN, TIAM, TYL, AMX and PEN G was conducted by an UHPLC Shimadzu Nexera X2 (Shimadzu, Kyoto, Japan) system connected to the SCIEX 4500 triple quadrupole mass spectrometer (Sciex, Framingham, MA, USA) controlled by Analyst 1.6.2 software (SCIEX, Framingham, MA, USA). The mass spectrometry detection was operated in the positive ESI mode with multiple reaction monitoring (MRM). The operating parameters were set as follows: temperature—450 °C, curtain gas (N_2_)—20; nebulizer gas (N_2_)—60; collision gas (N_2_)—medium; auxiliary gas—65; ion spray voltage—4500 V. Mass spectrometric conditions are shown in Table 4.

The chromatographic separation assay was performed using an Agilent Zorbax SB (50 × 2.1 mm, 1.8 µm) column (Agilent, St Clara, CA, USA) with an octadecyl guard column (2 × 4 mm) maintained at 35 °C. The mobile phase consisted of 0.025% heptafluorobutyric acid (A) and acetonitrile (B) at a flow rate of 0.6 mL/min with an injection volume of 5 µL. Gradient elution was conducted as follows: 0–4 min 90% A, 4–5.3 min 20% A and finally from 5.31 to 7 min back to 90% A. The total run time was set as 7 min.

### 4.3. Method Validation

The method was validated according to the Commission Decision 2002/657/EC [31]. The following parameters were established: linearity, selectivity, precision (repeatability and within-laboratory reproducibility) and recovery. In addition, LOD and LOQ were estimated (EUR 28,099 EN) [32]. The linearity in the matrix of oral fluid was checked by preparing two calibration curves at six different spiked levels and at low concentration ranges: 0, 0.5, 1.0, 1.5, 2.0, 5.0× validation level (VL), as well as high concentrations 10, 20, 50, 80, 100, 120× VL. VLs were different depending on the analyte, and the following levels were set: VL = 100 µg/L for TIAM, LIN, SDX, TRMP, TYL and VL = 10 µg/l for PEN G and AMX. The linearity was calculated as the squares linear regression by plotting the analyte/internal standard area ratio response versus the analyte/internal standard added concentration. The selectivity was evaluated by analysing different origin oral fluid samples (*n* = 20) and checked for potential interferences with endogenous substances. The precision for each analyte was determined by the repeated analysis (*n* = 6) of oral fluid samples spiked with SDX, TRMP, TYL, TIAM, LIN, AMX and PEN G at three concentrations corresponding to 0.5, 1.0, 1.5× VL. The precision was calculated and expressed as the percentage coefficients of variation (CV, %). For repeatability, samples were analysed on the same day by the same operator. For within laboratory reproducibility, another two sets of fortified samples at the same concentration levels as for the repeatability were analysed on two different days with different operators. The extraction recovery experiment was carried out at the three concentration levels, in the same experiment as precision, by comparing the average area obtained for each analyte with the concentrations of analytes in spiked samples. Additionally, LOQ as the lowest point of the matrix-matched calibration curve, where the least abundant diagnostic ion for quantitative analysis can be detected was determined. The LOD was estimated as the signal to noise ratio (S/N) of 3, and LOQ was determined as the lowest validated concentration that produced a signal to noise ratio > 10.

## 5. Conclusions

The results of conducted experiment illustrate that oral fluid can be used to monitor most antibacterial substances administered in pig farms. This paper shows the usefulness of oral fluid as a biological material for the non-invasive detection of drugs administered on pig farms. An analytical UHPLC-MS/MS method for the simultaneous determination of seven antibacterials in the oral fluid has been developed and validated. The implemented method is suitable for the simultaneous rapid detection and quantitation of SDX, TRMP, TYL, TIAM, LIN, AMX and PEN G in porcine oral fluid. The concentrations of these compounds found in this work showed high distribution to oral fluid for SDX, TRMP, TYL, TIAM and LIN, except for AMX and PEN G, where low penetration was observed.

Analysis of antibiotics in oral fluid offers a cost-effective approach for the screening of an individual animal or populations of pigs. The results of this study demonstrate the oral fluid analysis as an important “security tool” for the rapid verification of the declaration of treatment on pig farms.

## Figures and Tables

**Table 1:** Validation parameters.

Analyte	Repeatability (CV, %)	Reproducibility (CV, %)	Recovery (%)	LOD (µg/L)	LOQ (µg/L)
Sulfadoxine	6.39 ± 1.3	12.7 ± 2.6	87.6 ± 6.6	2	5
Trimetophrim	8.92 ± 1.1	11.45 ± 2.4	97.3 ± 4.4	2	5
Lincomycin	7.07 ± 1.5	13.2 ± 2.9	94.2 ± 4.2	1	2
Tiamulin	4.66 ± 1.1	10.1 ± 2.2	106 ± 5.6	1	2
Tylosin	9.59 ± 1.6	14.2 ± 3.1	87.9 ± 4.9	2	5
Amoxycyllin	9.55 ± 1.8	14.9 ± 3.8	107 ± 4.6	5	10
Penicillin G	9.91 ± 1.9	12.0 ± 2.7	101 ± 4.1	2	5

**Table 2:** Results of oral fluid samples analysis.

	Avarage Concentrations (µg/L)							
Period	Time (d)	SDX + TRMP		LIN	TIAM	TYL	AMX	PEN G
Treatment	1	22,300 ± 3555	14,100 ± 2556	10,500 ± 1889	7600 ± 1055	396 ± 68	11.3 ± 1.6	93 ± 21
	2	5860 ± 1155	2810 ± 556	2960 ± 446	2520 ± 355	958 ± 166	15.2 ± 2.3	50 ± 15
	3	4570 ± 766	1810 ± 311	4250 ± 559	2460 ± 311	832 ± 142	14.6 ± 1.8	11 ± 4.6
Posttreatment	4	4010 ± 601	1030 ± 221	1550 ± 211	1560 ± 225	654 ± 111	11.4 ± 1.4	16 ± 5.4
	5	5780 ± 788	1400 ± 255	1674 ± 199	1540 ± 233	538 ± 96	11.2 ± 1.6	5.2 ± 1.1
	6	4070 ± 669	1280 ± 198	1790 ± 203	2960 ± 299	475 ± 89	<LOQ	52 ± 13
	7	310 ± 98	264 ± 73	1480 ± 186	2570 ± 254	175 ± 54	<LOQ	32 ± 7.2
	8	392 ± 88	125 ± 41	437 ± 101 *	875 ± 156	118 ± 41	<LOQ	6 ± 1.2
	9	386 ± 72	128 ± 38	457 ± 96	567 ± 111	158 ± 62	<LOQ	<LOQ *
	10	134 ± 49	36.4 ± 15	420 ± 83	621 ± 124	49.3 ± 13	<LOQ	<LOQ
	11	247 ± 62 *	67.1 ± 20 *	194 ± 55	279 ± 66	54.4 ± 16	<LOQ *	<LOQ
	12	74.8 ± 21	25.2 ± 9.6	257 ± 76	254 ± 69	140 ± 36	<LOQ	<LOQ
	13	68.0 ± 16	22.0 ± 8.7	131 ± 41	138 ± 43 *	32.3 ± 11	<LOQ	<LOQ
	14	77.4 ± 18	22.1 ± 6.4	125 ± 33	269 ± 71	30.6 ± 8.4	<LOQ	<LOQ
	15	69.8 ± 14	31.6 ± 11	277 ± 61	156 ± 41	19.2 ± 5.2	<LOQ	<LOQ
	16	78.4 ± 12	24.2 ± 8.2	337 ± 69	279 ± 68	52.8 ± 33	<LOQ	<LOQ
	17	47.0 ± 18	18.9 ± 4.8	128 ± 28	182 ± 49	31.9 ± 10	<LOQ	<LOQ
	18	44.2 ± 16	13.0 ± 3.6	302 ± 51	176 ± 52	13.9 ± 3.6	<LOQ	<LOQ
	19	58.4 ± 11	42.0 ± 14	279 ± 44	202 ± 45	16.1 ± 4.6	<LOQ	<LOQ
	20	76.4 ± 14	36.4 ± 10	376 ± 52	133 ± 36	25.4 ± 7.7	<LOQ	<LOQ
	21	21.4 ± 7.3	10.2 ± 4.4	252 ± 39	148 ± 32	9.2 ± 2.1	<LOQ	<LOQ
	22	25.3 ± 6.9	46.4 ± 16	142 ± 24	220 ± 44	5.5 ± 1.1	<LOQ	<LOQ
	23	15.9 ± 7.2	6.9 ± 2.4	298 ± 33	146 ± 29	5.4 ± 0.8	<LOQ	<LOQ
	24	37.0 ± 5.4	19.4 ± 5.5	326 ± 47	310 ± 55	6.6 ± 0.9 *	<LOQ	<LOQ
	25	31.5 ± 6.3	17.8 ± 4.1	180 ± 44	193 ± 33	5.0 ± 1.3	<LOQ	<LOQ
	26	19.1 ± 4.2	15.9 ± 3.6	160 ± 36	66.4 ± 12	3.0 ± 0.5	<LOQ	<LOQ
	27	19.3 ± 3.7	10.4 ± 2.9	205 ± 43	54.3 ± 14	6.8 ± 1.7	<LOQ	<LOQ
	28	35.0 ± 8.1	16.5 ± 6.2	103 ± 26	35.8 ± 17	<LOQ	<LOQ	<LOQ
	29	30.4 ± 7.6	21.2 ± 9.4	85.6 ± 16	40.6 ± 21	<LOQ	<LOQ	<LOQ
	30	23.0 ± 6.2	22.4 ± 9.1	58.1 ± 12	46.9 ± 19	<LOQ	<LOQ	<LOQ

LOQ—limit of quantification. *—withdrawal period for each compound.

**Table 3:** Drugs and dosage administered to animals.

Administered Drug	Active Substance	Group of Antibacterials	Withdrawal Period for Tissues (Days)	Dosage (Active Substance Per 1 kg of Body Weight)	Practical Dosage
Borgal 24%	trimethoprim	diaminopyrimidines	8	3 mg of trimethoprim and 12 mg of sulfadoxine	1 mL for 16 kg, 2× with 48 h interval
	sulfadoxine	sulfonamides			
Biotyl 200	tylosin	macrolide	21	5 mg	1 mL for 40 kg, 3× with 24 h interval
Lincomycin VMD	lincomycin	lincosamides	5	10 mg	1 mL for 10 kg, 3× with 24 h interval
Probencil	penicillin G	penicillins	6	10 mg	1 mL for 30 kg, 3× with 24 h interval
Tiamowet 200	tiamuline	pleuromutilins	10	6 mg	1 mL for 20 kg, 3× with 24 h interval
Vetrimoxin LA	amoxicillin	penicillins	8	15 mg	1 mL for 10 kg, 2× with 48 h interval

**Table 4:** MS/MS parameters and summary of the MRM monitored for presented analytes.

Analyte	Parent Ion (*m*/*z*)	Daughter Ion(s) (*m*/*z*)	CXP (V)	DP (V)	CE (eV)	Dwell Time (msec)
SDX	310.9	156; 108	13	60	25; 40	50
TRMP	292.1	262; 231	5	52	36; 33	70
LIN	407.2	126; 359	7	74	36; 28	50
TIAM	494.4	192; 119	18	128	30; 56	50
TYL	916	174; 772	20	110	52; 42	50
AMX	366.1	349; 208	8	45	12; 18	100
PEN G	335.1	160; 176	12	60	17; 48	100
SFF (IS)	315.1	156	15	90	26	50
LIN d_3_ (IS)	410.0	129	13	66	44	50
AMX d_4_ (IS)	371.0	354	13	35	15	50
AZT (IS)	759.0	591	13	89	40	50

## Data Availability

Data is contained within the article.
